# A case of complete response following the administration of pembrolizumab and metastasectomy for lung and bone metastases of bladder cancer

**DOI:** 10.1002/iju5.12402

**Published:** 2021-12-15

**Authors:** Takehiko Nakasato, Tatsuki Inoue, Ryosuke Kato, Yoshihiro Nakagami, Kazuhiko Oshinomi, Yoshiko Maeda, Jun Morita, Takeshi Shichijo, Toshiko Yamochi, Takashi Fukagai

**Affiliations:** ^1^ Departments of Urology Showa University School of Medicine Tokyo Japan; ^2^ Pathology Showa University School of Medicine Tokyo Japan

**Keywords:** bladder cancer, complete response, metastasectomy, pembrolizumab, urothelial cancer

## Abstract

**Introduction:**

Patients with metastatic urothelial carcinoma have poor prognosis and limited treatment options.

**Case presentation:**

The patient was a 60‐year‐old male with bladder cancer and multiple lung metastases. He underwent three courses of gemcitabine and cisplatin chemotherapy, despite left femoral bone metastases. Tumor resection and bone replacement surgery was performed. Following the administration of four courses of pembrolizumab, lung metastasis completely resolved. However, after nine courses, right femoral neck bone metastasis was observed; therefore, tumor resection and bone replacement surgery were repeated. Pathologically, PD‐L1 expression was low in lung biopsy tissue and bone metastases. Pembrolizumab treatment continued for up to 20 courses; cancer recurrence and adverse events were not observed upon follow‐up examination after 1 year.

**Conclusion:**

Patients responding well to systemic therapy may have resectable metastatic sites, and long‐term survival might be achieved with adjunctive metastasectomy. The effect of pembrolizumab was not associated with positive PD‐L1 expression.

**Abbreviations & Acronyms**
CPS: combined positive score
CR: complete response
ICIs: immune checkpoint inhibitors
OS: overall survival
PD‐1: programmed death‐1
PD‐L1: programmed death‐ligand 1
TURBT: transurethral resection of the bladder tumor
UC: urothelial carcinoma

**Keynote message**

The therapeutic response to pembrolizumab varies depending on the site of metastasis. Cases that show complete response upon metastasectomy of resectable sites should be considered for this therapy. Pembrolizumab may be effective even if the programmed death‐1 antibody tests are negative.

## Introduction

Patients with metastatic UC have a poor prognosis and limited treatment options. As the standard first‐line treatment, platinum‐based combination chemotherapy is associated with a median OS of 12–15 months.^1^ However, platinum‐based chemotherapy has been reported to be ineffective against bone metastases of UC, and hence, confers a poor prognosis.^2^

In the KEYNOTE‐045 Clinical Trials, the administration of pembrolizumab, a monoclonal antibody against PD‐1, and chemotherapy with paclitaxel, docetaxel, or vinflunine, was used as a second‐line therapy for patients with metastatic UC. However, the prognostic prolongation effect was limited, with an OS of 10.3 and 7.4 months observed in the pembrolizumab and chemotherapy groups, respectively.^3^

The suitable response rate of ICIs is generally 20%, with a complete response of approximately 2–7%. Unfortunately, prolonged responses to ICIs alone are rare, and many patients experience uncontrolled disease responses.^4^, ^5^ However, we occasionally encounter patients who respond well to systemic therapy for some metastases but not for certain isolated sites; therefore, their treatment cannot be considered successful. For such patients, additional metastasectomy may be a potential treatment option.

## Case presentation

**Fig. 1:**
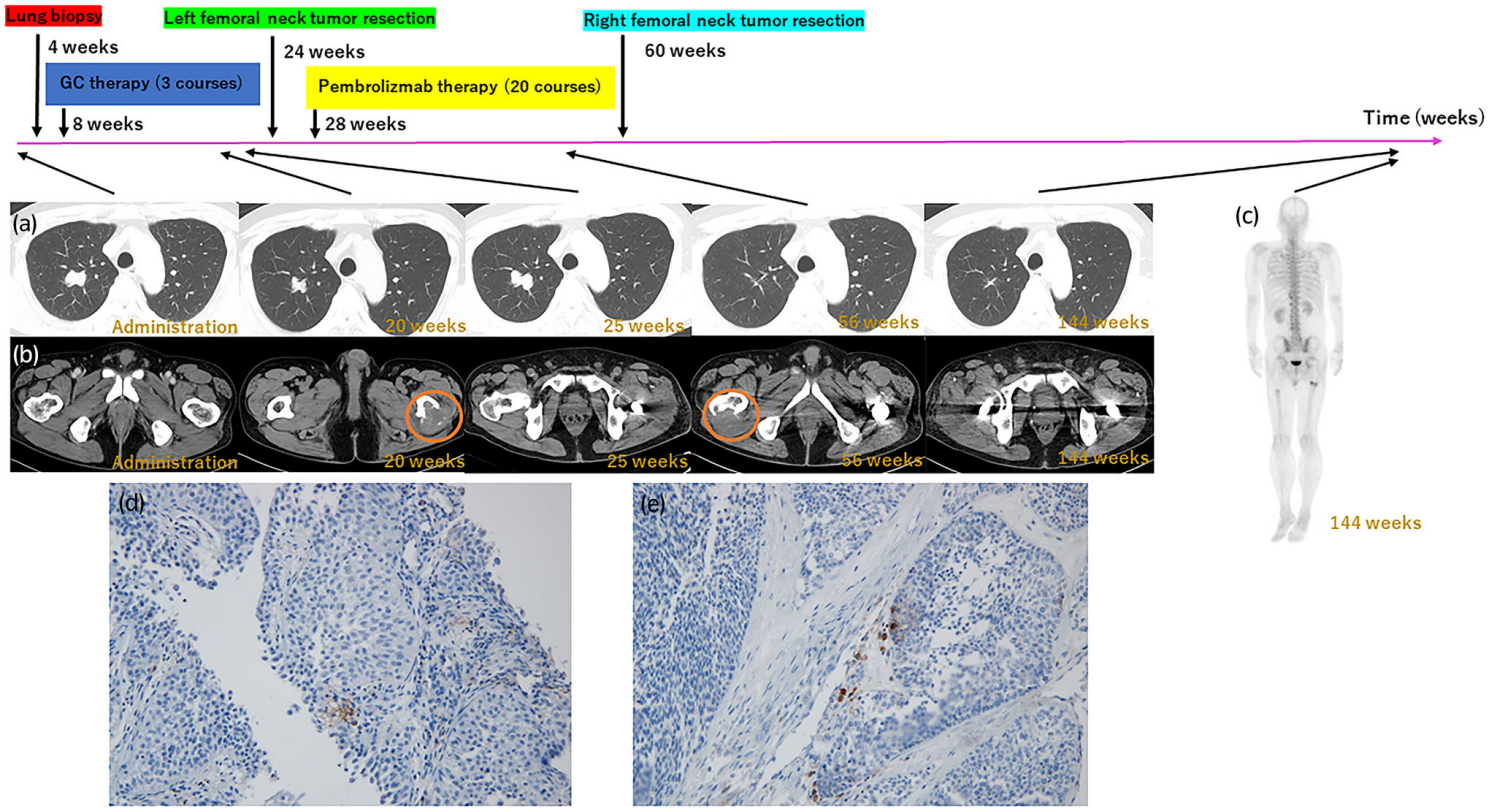
(a) Clinical course of lung metastasis. (b) Clinical course of femoral metastasis. Orange circle shows the appearance of new lesions. (c) No bone metastasis at 144 weeks. (d) Pathological findings of PD‐1 staining of the lung metastases. (e) Pathological findings of PD‐1 staining of the bone metastases. PD‐1, programmed death 1.

The patient was a 60‐year‐old male with bladder cancer (cT2aN0M1) that progressed to multiple lung metastases (Fig. 1a). The condition of the patient at the time of admission indicated the probability of primary lung cancer. Therefore, the patient underwent TURBT and lung biopsy. Pathological examination after TURBT confirmed the presence of high‐grade UC (pT2), and lung tumor biopsy confirmed metastases of UC. The patient did not undergo local treatment after TURBT.

Three courses of GC chemotherapy (gemcitabine 1000 mg/m^2^, days 1, 8, and 15; cisplatin 70 mg/m^2^, day 2) were administered for metastatic UC, which resulted in the decrease of lung metastases (Fig. 1a). However, occurrence of a bone metastatic (left femoral neck) tumor was observed following the chemotherapy (Fig. 1b). The left femoral neck was replaced after resection of the metastatic tumor. A clean surgical margin was confirmed by pathological examination. During the resection procedure, lung metastases increased due to drug withdrawal (Fig. 1a). Subsequent administration of four courses of pembrolizumab resulted in complete eradication of the lung metastasis. As the administration of pembrolizumab continued for nine courses, a new bone metastatic tumor (right femoral neck) appeared (Fig. 1a,b); hence, right femoral neck tumor resection and bone replacement were repeated. The surgical margins were then cleaned and confirmed by pathological examination. Thereafter, pembrolizumab administration was continued for up to 20 courses, with no recurrence or adverse events. Pembrolizumab was discontinued once the patient showed CR to avoid potential side effects. Two years and 11 months after initiating treatment for metastatic UC, CR was maintained (Fig. 1a–c), and we confirmed that no local recurrence was visually detected using a cystoscope.

The lung tumor biopsy site and excised bone metastasis specimen were stained with PD‐L1 IHC 22C3 pharmDx, revealing a CPS of <1%, indicating low PD‐L1 expression (Fig. 1d,e). CPS can be calculated using the following equation: CPS = Number of PD‐L1 staining cells (tumor cells, lymphocytes, macrophages)/total number of viable tumor cells × 100.

## Discussion

In the era of cytotoxic chemotherapy, several studies have attempted to identify the specific group of patients who can achieve maximum benefit from metastasectomy. It was found that patients with low‐volume and chemotherapy‐sensitive metastatic disease, confined to the lungs or lymph nodes, may be the best candidates for incorporating metastasectomy as a treatment option. ^6^, ^7^, ^8^, ^9^, ^10^

Moreover, the extent of disease in such patients may contribute to favorable underlying disease biology, and theoretically have favorable outcomes, even without metastasectomy. Based on a systematic review and meta‐analysis, survival after metastasectomy for metastatic UC has been reported, wherein a subset of patients who underwent metastasectomy achieved stability; however, the current level of evidence precludes the establishment of more general recommendations.^11^

Only 2–7% of cases obtain CR by ICI alone, whereas many cases could not achieve CR. There have been a few reports on metastasectomy with ICI therapy, but further research is required for confirmation. However, the role of metastasectomy may need to be re‐envisioned, since modulation of the host’s immune system may alter the course of disease and patterns of progression in ways that are uncommon in the era of cytotoxic chemotherapy. In this case, bone metastasis appeared during treatment with chemotherapy and monoclonal antibody against PD‐1. Consequently, the patient developed a progressive disease. Regarding lung metastasis, contrary to the progression of bone metastasis, tumor disappearance was observed following the administration of monoclonal antibody against PD‐1, and CR was obtained with metastasectomy for bone metastasis. The stage of metastatic UC may range from a systemic disease that is difficult to treat oligo‐metastasis that may be treated curatively depending on the treatment method. Even if pembrolizumab alone does not provide an adequate therapeutic effect, CR may be achieved if the metastatic lesion is resectable. However, it is important to consider whether tumor resection remains solely a cytoreductive therapy, as the disease may lose control eventually.

The PD‐1 antibody positivity rate in this case was low for both lung metastases, wherein the administration of pembrolizumab resulted in high reactivity, and bone metastases were poorly reactive. Even in the KEYNOTE‐045 Clinical Trials, superiority has not been verified for progression‐free survival in patients with high PD‐1 antibody expression.^3^ It may be difficult to predict the therapeutic effect of pembrolizumab based on the positivity rate of PD‐1 antibody in UC.

## Author contribution

Takehiko Nakasato: Conceptualization; Data curation; Investigation; Methodology; Project administration; Writing – original draft. Tatsuki Inoue: Project administration. Ryosuke Kato: Project administration. Yoshihiro Nakagami: Project administration. Kazuhiko Oshinomi: Project administration. Yoshiko Maeda: Project administration. Jun Morita: Project administration. Takeshi Shichijo: Project administration. Toshiko Yamochi: Supervision; Validation. Takashi Fukagai: Project administration; Supervision; Writing – review & editing.

## Conflict of interest

None declared.

## Approval of the research protocol by an Institutional reviewer board

The study was approved by the Ethics Committee of Showa University Hospital (Approval numbers: 3383).

## Informed consent

The patient involved provided informed consent for the publication of this study.

## Registry and the registration no. of the study/trial

Not applicable.
